# Analysis of 2 men with t(8;22)(q13;q13) and t(8;14)(q13;q22) chromosomal translocation karyotypes

**DOI:** 10.1097/MD.0000000000031091

**Published:** 2022-10-14

**Authors:** Qijia Sun, Xiaoyu Zhang, Peng Zhan, Wenjie Tian, Yanli Wang, Xiao Yang

**Affiliations:** a Department of Urology, The Second Hospital of Jilin University, Changchun, China.

**Keywords:** breakpoint, genetic counseling, male infertility, reciprocal chromosomal translocation

## Abstract

Male infertility is a multifactorial condition that is closely associated with chromosomal abnormalities. Reciprocal chromosomal translocation (RCT) is a significant structural genetic abnormality. The specific mechanisms of forms of RCT affecting male infertility include the product of chromosomally unbalanced gametes, thereby disrupting the structure and function of important genes responsible for spermatogenesis. RCT breakpoints have been found to disrupt gene structure and function in many medical fields However, the relationship between RCT breakpoints and male infertility remains to be determined. The purpose of this study is to describe 2 male carriers of RCTs 46,XY,t(8;22)(q13;q13) and 46,XY,t(8;14)(q13;q22). Both patients were collected from the second hospital of Jilin University. Semen parameters were detected using the computer-aided semen analysis system. Cytogenetic analysis was performed using standard operating procedure. Related genes on chromosomal breakpoints were searched using Online Mendelian Inheritance in Man. One man had semen parameters within the normal range, but the couple was infertile after 5 years of marriage. The other man showed normal semen parameters, and his wife had experienced 2 spontaneous miscarriages. Using a literature search, the association between chromosome 22q13 breakpoint and fertility were investigated. The results suggest that physicians should focus on the clinical phenotype of the patients and the breakpoints of RCT in genetic counseling. An important gene related to human male infertility is clearly located in chromosome region 22q13, and its function is worthy of further study.

## 1. Introduction

Male infertility is a multifactorial condition, and it is closely associated with chromosomal abnormalities.^[1,2]^ Reciprocal chromosomal translocations (RCTs) are genetic abnormalities observed in 0.4% to 1.4% of infertile men and are considered to be a cause of male factor infertility.^[3,4]^ RCT carriers produce chromosomally unbalanced gametes, which increase the risk of recurrent spontaneous abortion.^[5]^ Moreover, RCT breakpoints can disrupt the structure and function of important genes responsible for spermatogenesis, and men affected by these can exhibit azoospermia, oligozoospermia, asthenozoospermia or teratospermia.^[4,6,7]^ However, the underlying pathological mechanisms remain unclear.

RCT breakpoints have been found to disrupt gene structure and function in many medical fields. Yeates et al^[8]^ reported that an RCT disrupting the sodium voltage-gated channel *α*-subunit 5 gene is associated with Brugada syndrome and sudden cardiac death. Peng et al^[9]^ reported that an RCT breakpoint disrupting the tumor protein p63gene is related to split hand/foot malformation. Cacciagli et al^[10]^ reported that a de novo t(10;13) balanced translocation disrupts the coding sequence and expression of the *ATPase, class I, type 8A, member 2* gene, which causes a phenotype of mental retardation in humans. Wang et al^[11]^ reported that several RCT breakpoints disrupt genes (*Nucleoporin, 155-KD, Fibronectin type III domain-containing protein 3A* and *Dpy19-like 1*) related to male infertility. Chen et al^[12]^ also reported that some RCT breakpoints can disrupt important genes involved in spermatogenesis. Although some RCT breakpoints have been reviewed,^[13,14]^ the relationship between other RCT breakpoints and infertility remains to be determined.

The aim of this study was to identify clinical features of 2 RCTs at t(8;22) and t(8;14), and to explore the relationship between such breakpoints and male infertility.

## 2. Materials and Methods

### 2.1. Patients

This study was approved by the Ethics Committee of the Second Hospital, Jilin University, P.R. China. Written informed consent has been obtained from both participants for the publication of these cases. The subjects of this study were 2 male carriers of RCTs. Both patients had visited the Andrology outpatient department of the hospital. A questionnaire was completed by each patient, including age, marriage status, pregnancy history, genetic family history, anamnesis information, smoking and drinking history, and any use of drugs. A general physical examination was performed to record each patient’s height, weight, growth and development information, and testicular size.

Case 1 involved an apparently normal 29-year-old man. He visited the hospital for medical consultation because of infertility after 5 years of marriage. Case 2 involved a 27-year-old male who presented to the andrology department because his wife had experienced 2 spontaneous miscarriages. Semen analysis and karyotyping were recommended for both patients. The clinical diagnosis and treatment information of both wives was collected, and karyotyping was also recommended. After both men were found to carry abnormal chromosomes, their parents were contacted for karyotyping.

### 2.2. Semen analysis

For each patient, semen analysis was performed using standard techniques recommended by the World Health Organization guidelines.^[15]^ Semen samples were obtained by masturbation after 3 to 7 days of abstinence, collected in a sterile container and examined after liquefaction by 2 professional technicians. Semen parameters were detected using a computer-aided semen analysis system (Beion S3, Shanghai Beion Medical Technology Co., Ltd, Shanghai, P.R. China).

### 2.3. Cytogenetic analysis

For each patient and his family, peripheral blood (2 mL) was collected in sterile tubes containing heparin anticoagulant. Lymphocytes were cultured in RPMI-1640 culture medium with 15% fetal bovine serum and stimulated by 2% phytohaemagglutinin (Yishengjun; Guangzhou Baidi Biotech, Guangzhou, China) for 72 hours at 37 °C. Then, G-banding was performed using standard procedures. The karyotypes were described according to the International System for Human Cytogenetic Nomenclature (ISCN 2020).

## 3. Results

### 3.1. Clinical profile

**Figure 1.**
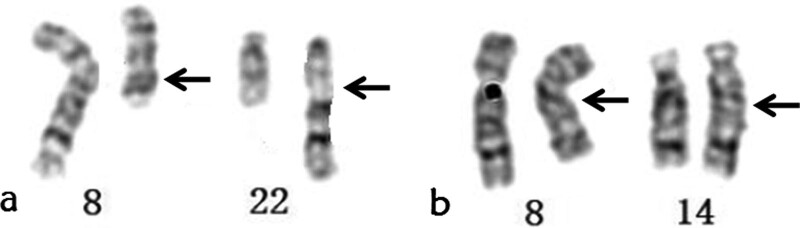
G-banding karyotypes of 2 patients in this study.

The clinical profile of both patients is shown in Table 1. Cytogenetic analysis revealed that the karyotype of Case 1 was 46,XY,t(8;22)(q13;q13) (Fig. 1A) and that of Case 1 was 46,XY,t(8;14)(q13;q22) (Fig. 1B). The karyotype of Case 1 had arisen de novo, while the karyotype of Case 2 had been inherited from his father. Semen parameters for both men were within the normal reference range.

**Table 1:** Clinical profile of both patients.

Item	Case 1	Case 2
Karyotype	46,XY,t(8;22)(q13;q13)	46,XY,t(8;14)(q13;q22)
Semen volume (mL)	1.8	2.0
Sperm concentration (10^6^ per mL)	16	22
Total motility (%)	42	46
Progressive motility (%)	35	38
Sperm morphology (normal forms, %)	5	10
Karyotype of spouse	46,XX	46,XX
Routine genomic examination of spouse	No abnormal changes were observed.	No abnormal changes were observed.
Paternal karyotype	46,XY	46,XY,t(8;14)(q13;q22)
Maternal karyotype	46,XX	46,XX

### 3.2. Analysis of translocation breakpoints

Three breakpoints were involved in these 2 cases. Both of these RCT carriers exhibited a chromosome 8q13 breakpoint. Case 1, with a chromosome 22q13 breakpoint, showed infertility after 5 years of marriage. The wife of Case 2 with a chromosome 14q22 breakpoint had a history of 2 spontaneous abortions. To clarify the relationship between these breakpoints and the clinical phenotypes, genes and loci associated with infertility or sperm function were searched using Online Mendelian Inheritance in Man (https://www.ncbi.nlm.nih.gov/omim/). We identified that 9 important genes and their functions are associated with RCT breakpoints (Table 2). Of these, the chromosome 22q13 breakpoint is closely linked to impaired reproductive function or male infertility.

**Table 2:** The genes and their functions associated with translocation breakpoints.

Gene*	Full name	Loci	Expression or Function
*MCMDC2*	Minichromosome maintenance domain-containing protein 2	8q13.1	Expressed in testis, particularly in spermatocytes
*AP5M1*	Adaptor-related protein complex 5,mu-1 subunit	14q22.1	Highly expressed in maturing sperm cells
*PICK1*	Protein interacting with C kinase 1	22q13.1	PICK1 deficiency causes male infertility in mice by disrupting acrosome formation
*DMC1*	DNA meiotic recombinase 1	22q13.1	Have a possible association between variation in the DMC1 gene and azoospermia
*HYDM3*	Hydatidiform mole, recurrent, 3	22q13.2	Recurrent hydatidiform mole-3 is caused by homozygous or compound heterozygous mutation in the MEI1 gene
*MEI1*	Meiotic double-stranded break formation protein 1	22q13.2	Polymorphic alleles of the human MEI1 gene are associated with human azoospermia by meiotic arrest
*ACR*	Acrosin	22q13.33	Men with reduced acrosin activity in their spermatozoa have fertility problems
*RABL2B*	RAB, member of Ras oncogene family-like 2b	22q13.33	Have redundant roles in intraflagellar transport and ciliogenesis
*MOV10L1*	MOV10-like 1	22q13.33	MOV10L1 is an RNA helicase that functions in the biogenesis of piRNAs

* From: https://www.ncbi.nlm.nih.gov/omim/

To further explore the relationship between chromosome 22q13 breakpoint and infertility, a literature search was performed that identified 18 carriers. The karyotypes and clinical features of these cases were collected and are summarized in Table 3. Almost all the female carriers exhibited recurrent spontaneous abortions. However, the clinical manifestations of male carriers are varied. Of these male individuals, 3 chose to use intracytoplasmic sperm injection technology for conception, and a carrier with t(11;22)(q22.3;q13.3) had good semen quality.

**Table 3:** Karyotypes and clinical features of carriers with a chromosome 22q13 translocation.

Case	Sex	Karyotype	Clinical Features	Reference
1	M	t(1;22)(q41;q13)	ICSI	Gekas et al^[16]^
2	M	t(6;22)(q13;q13)	N/A	Anton et al^[17]^
3	M	t(6;22)(q25.3;q13.31)	Fair semen quality, ICSI	Mayeur et al^[18]^
4	M	t(10;22)(q25;q13)	Stillbirth	Li et al^[19]^
5	M	t(10;22)(q25;q13)	Spontaneous abortion	Zhang et al^[20]^
6	M	t(11;22)(q22.3;q13.3)	Good semen quality, ICSI	Mayeur et al^[18]^
7	M	t(11;22)(q23;q13)	Recurrent abortion	Gaboon et al^[21]^
8	M	t(11;22)(q25;q13)	Asthenospermia	Zhang et al^[20]^
9	M	t(15;22)(q22;q13)	PGD	Escudero et al^[22]^
10	M	t(15;22)(q13;q13.3)	PGD	Pundir et al^[23]^
11	F	t(2;22)(p22;q13.2)	Spontaneous abortion	Ikuma et al^[24]^
12	F	t(6;22)(q26;q13.32)	Abnormal offspring	Manvelyan et al^[25]^
13	F	t(7;22)(q11;q13)	Spontaneous abortion	Bourrouillou et al^[26]^
14	F	t(11;22)(q25;q13)	PGD	Gianaroli et al^[27]^
15	F	t(11;22)(q25;q13)	Spontaneous abortion	Husslein et al^[28]^
16	F	t(11;22)(q25;q13)	Repeated miscarriage	Iyer et al^[29]^
17	F	t(11;22)(p11;q13)	Recurrent abortion	Portnoï et al^[30]^
18	F	t(X;22)(p11.21;q13.3)	Recurrent miscarriage	Dutta et al^[31]^

ICSI = intracytoplasmic sperm injection, N/A = not applicable, PGD = preimplantation genetic diagnosis.

## 4. Discussion

RCT is a significant structural genetic abnormality that is closely related to male infertility.^[12]^ In clinical practice, the phenotypic characteristics of male RCT carriers are varied. Some carriers could have offspring with normal phenotypes without any fertility problems.^[13]^ Others were diagnosed with abnormal sperm parameters.^[4,14,32,33]^ During spermatogenesis, spermatogenic cells complete meiosis. For translocation carriers, the 2 translocated chromosomes and their 2 homologous normal chromosomes form a quadrivalent and subsequently segregate at anaphase I. For multiple meiotic segregation patterns: alternate segregation involves 2 normal non-homologous chromosomes and 2 translocated chromosomes that migrate to different spindle poles; adjacent-1 or -2 segregated, homologous centromeres pass to the opposite or the same spindle poles, respectively; in 3:1 or 4:0 segregation, 3 or 4 out of the 4 chromosomes move to 1 pole, with the remaining chromosome(s) moving to the other 1.^[34]^ Spermatozoa produced by adjacent-1 or -2 segregation and 3:1 or 4:0 segregation patterns have unbalanced chromosomes. Hence, the wives of these carriers showed recurrent spontaneous abortion.^[35,36]^ Therefore, genetic counseling remains a challenge for RCT carriers. Here we report 2 men carrying 46,XY,t(8;22)(q13;q13) and 46,XY,t(8;14)(q13;q22) karyotypes. Semen parameters of the former were normal but the couple was infertile for 5 years after marriage. The wife of the latter had recurrent miscarriages.

Coincidentally, both patients carried chromosome 8q13 breakpoints, but their clinical phenotypes were different. Therefore, breakpoints of 22q13and 14q22 are worthy of further study. Through a gene search in Online Mendelian Inheritance in Man, the relationship between these breakpoints and infertility were reviewed. The *Minichromosome maintenance domain-containing protein 2* gene is located on chromosome 8q13.1 and is expressed in the testis, particularly in spermatocytes.^[37]^ *Adaptor-related protein complex 5,mu-1 subunit*, mapping to chromosome 14 at 14q22.1 is highly expressed in maturing sperm cells.^[38]^ The *Protein interacting with C kinase 1* gene located on chromosome 22q13.1 could disrupt acrosome formation, causing male infertility.^[39]^ The Acrosin gene is located on chromosome 22q13.33 and male carriers show reduced acrosin activity in their spermatozoa and have fertility problems.^[40]^ Houston et al^[41]^ reviewed the validated monogenic causes of human male infertility, and noted that the *meiotic double-stranded break formation protein 1* gene (meiotic double-stranded break formation protein 1) is located at breakpoint 22q13.2 and is associated with nonobstructive azoospermia. However, the cases in the present study have semen parameters within the normal range.

We further explored the relationship between the chromosome 22q13 breakpoint and infertility and identified 7 genes. Kim et al^[42]^ reported that chromosome 22q13 is associated with abnormal spermatogenesis. Hong et al^[4]^ reported a carrier with 46, XY, t(9;22) (q22;q13) who exhibited oligospermia and asthenospermia. Table 2 shows that the chromosomal 22q13 translocation is associated with high reproductive risks such as recurrent miscarriage, asthenospermia or abnormal offspring carrying a chromosomal imbalance. For female carriers, almost of all are related to recurrent spontaneous abortion. For male carriers, the clinical manifestations are varied. These results suggest that male RCT carriers inherited this from their mothers could be infertile. This phenomenon should be paid attention to in clinical genetic counseling. In addition, 1 case with t(11;22)(q22.3;q13.3) was similar to Case 1 in this study, and had good semen quality, which might be related to the abnormal Acrosin gene structure. The significance of this disruption remains to be determined, and can be used for further analysis. However, such individuals can choose intracytoplasmic sperm injection to increase the chances of pregnancy following clinical genetic counseling.

The limitation of this study is that there was no genetic analysis of the aborted tissues in Case 2. Therefore, clinicians should advise patients with recurrent abortion that aborted tissues should be collected for further testing to assist future genetic counseling.

## 5. Conclusions

In conclusion, we report 2 male carriers of RCT. The chromosome 22q13 breakpoint is associated with male infertility, and an important gene related to infertility is clearly located here. The function of this gene is worthy of further study. Taken together with the published literature, these results suggest that physicians should focus on the clinical phenotype of the patients and the RCT positions in genetic counseling.

## Acknowledgments

We thank James M Cummins, PhD, Liwen Bianji (Edanz) (www.liwenbianji.cn) for editing the language of a draft of this manuscript.

## Author contributions

**Conceptualization:** Qijia Sun, Peng Zhan.

**Data curation:** Qijia Sun, Xiaoyu Zhang.

**Investigation:** Peng Zhan, Qijia Sun, Wenjie Tian.

**Methodology:** Yanli Wang, Xiao Yang.

**Writing – original draft:** Qijia Sun.

**Writing – review & editing:** Peng Zhan.
